# Flaxseed intervention and reproductive endocrine profiles in patients with polycystic ovary syndrome: an open-labeled randomized controlled clinical trial

**DOI:** 10.3389/fendo.2025.1531762

**Published:** 2025-04-07

**Authors:** Zahra Najdgholami, Fatemeh Maleki Sedgi, Samira Sadat Ghalishourani, Marzieh Feyzpour, Mehran Rahimlou

**Affiliations:** ^1^ Department of Midwifery, Zanjan University of Medical Sciences, Zanjan, Iran; ^2^ Department of Nutrition, School of Public Health, Zanjan University of Medical Sciences, Zanjan, Iran; ^3^ Metabolic Diseases Research Center, Health and Metabolic Research Institute, Zanjan University of Medical Science, Zanjan, Iran

**Keywords:** flaxseed, polycystic ovary syndrome (PCOS), reproductive endocrine profile, follicle-stimulating hormone (FSH), nutrition

## Abstract

**Introduction:**

Polycystic Ovary Syndrome (PCOS), affecting 6–15% of women of reproductive age, is characterized by hormonal imbalances and metabolic disturbances. Flaxseed, rich in omega-3 fatty acids and phytoestrogens, may offer a natural approach to improve reproductive hormone profiles in PCOS patients. This study is aimed at evaluating the effects of flaxseed supplementation on reproductive endocrine profiles in women with PCOS.

**Methods:**

In this open-labeled, randomized controlled trial, 70 women with PCOS were randomly assigned to either the intervention group (n=35), receiving 30 grams of milled flaxseed daily along with lifestyle modifications, or the control group (n=35), receiving only lifestyle modifications, for 12 weeks. The primary outcome was the change in follicle-stimulating hormone (FSH) levels. Secondary outcomes included changes in serum concentrations of luteinizing hormone (LH), the LH/FSH ratio, anti-Müllerian hormone (AMH), estradiol, dehydroepiandrosterone sulfate (DHEAS), and androstenedione.

**Results:**

After 12 weeks, the flaxseed group showed a significant increase in FSH levels compared to the control group. FSH levels increased from 9.72 ± 11.95 µU/mL at baseline to 10.59 ± 12.14 µU/mL after the intervention (p = 0.027). The mean treatment effect was 0.87 (95% CI: 0.086 to 1.75). The LH/FSH ratio also significantly decreased in the flaxseed group (mean treatment effect: -0.341, 95% CI: -0.63 to -0.08, p = 0.031). No significant changes were observed in AMH, LH, estradiol, DHEAS, or androstenedione levels.

**Conclusion:**

Flaxseed supplementation may benefit women with PCOS by increasing FSH levels and decreasing the LH/FSH ratio, suggesting its potential as a complementary intervention for managing hormonal disturbances in PCOS. Further studies are needed to confirm these findings and elucidate the underlying mechanisms.

## Introduction

PCOS, a common endocrine condition in women of childbearing years, afflicts between 6 and 15 percent of this demographic (1, 2). It is primarily characterized by irregular menstrual cycles, hyperandrogenism, and polycystic ovarian morphology on ultrasound (3, 4). PCOS is associated with an increased likelihood of developing metabolic syndrome, type 2 diabetes, and cardiovascular disease (2, 5, 6). Although the exact cause of PCOS remains unclear, obesity and insulin resistance (IR) are believed to play significant roles in its pathogenesis (7).

Metabolic irregularities, characterized by heightened concentrations of LH, normal or diminished levels of FSH, and an increased LH/FSH ratio, are frequently observed in individuals with PCOS and can have lasting effects on their overall health (8). Also, a key feature of PCOS is hyperandrogenism, driven by elevated testosterone levels due to stimulation of ovarian theca cells (8).

PCOS treatment is currently symptomatic and prescribed to reduce insulin resistance, lower androgen levels, alleviate inflammatory symptoms, and regulate menstrual cycles. Pharmacological treatment includes contraceptive hormones and other insulin-sensitizing agents, such as metformin (9, 10). However, PCOS treatment should include lifestyle changes such as dietary adjustments, increased physical activity, and behavioral interventions (11). Finally, there is a growing interest in the use of medicinal herbs as a complementary or alternative therapeutic approach for women with PCOS (12, 13).

Nutritional intervention is emerging as a promising strategy for managing PCOS (14, 15). Omega-3 polyunsaturated fatty acids (PUFAs) have demonstrated a range of health advantages, including anti-inflammatory properties, increased insulin sensitivity, and improved ovulation (16–18). Flaxseed, a plant-based food, is abundant in beneficial nutrients such as α-linolenic acid, phytoestrogenic lignans, and fiber (19, 20). Due to lignan’s structural similarity to sex hormones, this substance could potentially reduce the production of estrogen and increase the levels of sex hormone-binding globulin (SHBG) in adipose tissue and the liver (21).

Despite existing research on the effects of flaxseed on some metabolic and hormonal parameters in women with PCOS (22, 23), there is limited data on its impact on FSH, the LH/FSH ratio, AMH, estradiol, DHEAS, and androstenedione in PCOS patients. Therefore, a recent systematic review and meta-analysis on flaxseed and sex hormones (21), concluded that more RCTs of flaxseed supplementation on sex hormones are necessary to elucidate their complex relationship. Hence, this study aims to evaluate the effects of flaxseed on reproductive endocrine profiles in women with PCOS.

## Materials and methods

### Trial design

The PICO study (flaxseed and its impact on markers of fertility) was a monocentric, randomized, open-labeled controlled clinical trial. The study was approved by the local ethics review board at Ahvaz Jundishapur University of Medical Sciences in Iran (IR.AJUMS.REC.1396.619). The study followed ethical standards set by the Declaration of Helsinki and Good Clinical Practice. Additionally, the study was registered with the Iranian Registry of Clinical Trials (www.irct.ir) under the identifier IRCT20120704010181N11 (Registration date: 2018-01-21). To ensure transparency and rigor in reporting the trial, the researchers adhered to the Consolidated Standards of Reporting Trials (CONSORT) guidelines for randomized clinical trials. Prior to enrollment, all participants provided written informed consent. Participant recruitment commenced on 7 April 2019 and concluded on 3 March 2022. The study was temporarily suspended for a seven-month period during the COVID-19 pandemic.

### Sample size

We used a randomized clinical trial sample size calculation formula, where type one (α) and type two (β) errors were set at 0.05 and 0.20, respectively (power = 80%). Based on a previous trial (2), we estimated that a minimum of 35 participants per group would be required to detect a significant difference in FSH levels between the intervention and control groups.

### Participants

Patients were recruited at their first diagnosis of PCOS at the Endocrinology Clinic of Golestan Hospital, Ahvaz, Iran, after agreeing to participate and signing the informed consent form. Diagnosis of PCOS was based on the Rotterdam criteria (24) and the updated criteria available at the time of study initiation (25). At least two of the following criteria were required for the diagnosis: (1) clinical and/or biochemical hyperandrogenism, (2) ovulatory dysfunction (oligomenorrhea, amenorrhea), and (3) polycystic ovarian morphology on ultrasound according to the updated criteria (4). All participants were premenopausal women aged ≥18 years. Before establishing a diagnosis of PCOS, they were screened for conditions with overlapping clinical manifestations, including congenital adrenal hyperplasia, androgen-secreting tumors, Cushing’s syndrome, thyroid disorders, and hyperprolactinemia, to ensure accurate diagnosis and study eligibility.

Patients were excluded if they had any of the following criteria: pregnancy; overlapping hyperandrogenism causes; current use of supplements such as vitamin E, omega-3, or vitamin D; following restrictive diets (e.g, ketogenic or weight-loss diets); chronic conditions (cancer, diabetes, kidney disease, hypertension, inflammatory disorders, and autoimmune disease); allergy to flaxseed or any herbal product; and current hormonal treatments, including the use of combined oral contraceptive pills (COCs) in the three months prior to enrollment (26).

### Trial procedure and randomization

Subjects were randomly allocated (1:1) to either the flaxseed or control groups for a three-month intervention. The first group (n=35) consumed 30 grams of brown milled flaxseed daily in conjunction with lifestyle modifications. The control group (n=35) received lifestyle modifications alone. For the lifestyle modification program, a series of general nutritional and physical activity recommendations were explained to all patients in both groups in the same manner. Bulk flaxseed was acquired and stored in a cool, dark environment prior to the commencement of the study.

Randomization assignment was carried out using computer-generated random numbers. To ensure balanced groups, age (<30 and ≥30 years) and BMI (<25 and ≥25 kg/m²) were utilized as stratification factors. A randomization schedule was generated by an independent statistician affiliated with the Ahvaz Jundishapur University of Medical Sciences in Ahvaz, Iran. This sequence was subsequently integrated into the REDCap platform for implementation (27). The randomized allocation sequence, participant enrollment, and group allocation were conducted by trained staff at the clinic. Upon confirmation of eligibility, participants were randomly assigned to groups. Due to the nature of the study, it was not possible to blind the patients and the research team, but the laboratory operators who performed the biochemical tests were blinded to the intervention and control groups.

A week before distribution to participants, the flaxseed underwent a milling process and was subsequently packaged into 30-gram portions. The nutritional composition of the flaxseed powder was determined by the Ahvaz Food and Nutrition Research Laboratory. Each 100-gram serving contained 465 kilocalories, 39 grams of fat, 21 grams of alpha-linolenic acid (ALA), 18 grams of protein, 29 grams of carbohydrates, and 28 grams of dietary fiber. To monitor any changes in nutrient content, subsequent analyses were conducted at three-week intervals over a 12-week period.

At the initial visit (baseline), participants provided demographic and clinical data. Subsequently, the intervention group received a three-week allotment of milled flaxseed. Participants in this group were instructed to incorporate flaxseed into their daily diet by adding it to salads, yogurt, or cold beverages. Participants followed a comprehensive lifestyle intervention during the trial period, which included both nutritional guidance and physical activity recommendations. The nutritional component of the program focused on the dietary guidance aligned with recommendations from the American Heart Association (28), while the physical activity recommendations aimed to patients physical activity, aiming for at least half an hour of moderate-intensity exercise on three or more days each week.

### Compliance

To improve adherence to the study protocol, participants in the flaxseed group received daily text message reminders to consume the flaxseed powder. Participant compliance was assessed by measuring the quantity of returned flaxseed powder. Participants who consumed less than 90% of the recommended flaxseed were not included in the final data analysis.

### Trial outcomes

The primary outcome of this study was to assess the variation in the blood concentration of FSH during the study period in the intervention and control groups. Secondary outcomes included evaluating the effect of flaxseed compared to control on changes in other hormones including LH, LH/FSH ratio, AMH, estradiol, DHEAS and androstenedione.

For biochemical evaluations, 10 cc of fasting blood were taken from all patients after 10–12 hours of fasting at 8–9 in the morning at baseline (the day before starting the intervention) and at the end of the 12-week intervention. All blood samples were preserved for future analysis by storing them at a temperature of -80 degrees Celsius in the Ahvaz Jundishapur University of Medical Sciences Biobank. AMH, androstenedione, FSH, LH, 17β-estradiol, DHEAS and other biochemical parameters levels were measured at two time points: baseline (pre-intervention) and at the end of the study (post-intervention, after three months).

Hormonal assessments, including FSH, LH, AMH, estradiol, androstenedione, and DHEAS, were performed at specific points in the menstrual cycle to account for physiological fluctuations. In ovulatory participants, blood samples were collected in the early follicular phase (days 2–5 of the menstrual cycle) at both baseline and post-intervention. For anovulatory participants, samples were collected at random time points but remained consistent for each individual across both assessments to minimize variability. These procedures align with current clinical guidelines for hormonal assessment in PCOS.

FSH and LH levels were quantified using an enzyme-linked immunosorbent assay (ELISA) kit (Monobind Inc., USA). 17β-estradiol levels were quantified using IMMULATE^®^ CLIA assays (Siemens Healthcare Diagnostics Products Ltd., Glyn Rhonwy, UK). AMH was evaluated using an ELISA kit (Beckman Coulter, France). DHEAS levels were determined using an ELISA kit (Labor Diagnostika Nord, Nordhorn, Germany), and androstenedione levels were measured using an ELISA kit (Siemens Healthcare Diagnostics Products Ltd., Glyn Rhonwy, UK).

Serum insulin values were determined by the use of available ELISA kit (DiaMetra, Milano, Italy) with inter- and intra-assay coefficient variances (CVs) of 3.5 to 5.1%, respectively. Enzymatic kits of Pars Azmun (Tehran, Iran) were used to evaluate fasting blood sugar (FBS), serum triglycerides, total-, LDL- and HDL cholesterol values.

Participants’ diets were recorded over a three-day period using a 24-hour recall method administered at baseline and after study completion. The 24-hour recall was conducted using a standardized form developed for this study, which has been provided as **Supplementary Data**. Participants provided detailed information about their food and beverage consumption over these periods, and the data were subsequently analyzed for macronutrient and micronutrient content using the Nutritionist IV software package (Version 3.5, Axxya Systems, Stafford, TX, USA). The software is designed to analyze dietary intake data, provide nutrient breakdowns, and assess compliance with dietary guidelines.

Physical activity levels were assessed using the Metabolic Equivalent of Task (MET), a standardized unit that quantifies the energy expenditure of various activities. MET values were calculated based on self-reported physical activity data, where one MET represents the energy cost of resting metabolic rate, and higher MET values indicate greater physical activity levels. This measurement allowed for a comparison of baseline and post-intervention activity levels between the study groups.

### Statistical analysis

Data analysis was conducted using SPSS software (Version 20). Descriptive statistics were calculated for all variables, and the Kolmogorov-Smirnov test was employed to assess data distribution. Normally distributed continuous data were expressed as mean plus or minus standard deviation, while skewed data were presented as median with interquartile range. To assess differences between the groups at the study’s outset, numerical variables were analyzed using Student’s t-test, while categorical variables were compared with the chi-square test. In line with CONSORT recommendations for non-inferiority studies, both intention-to-treat (ITT) and per-protocol (PP) analyses were performed to evaluate the primary and secondary outcomes (29). Analysis of covariance (ANCOVA) was employed to assess differences in outcome variables between the flaxseed and control groups at study conclusion, with baseline values included as covariates to control for potential confounding effects. For all analyses, a p-value below 0.05 was deemed statistically significant.

## Results

**Figure 1:**
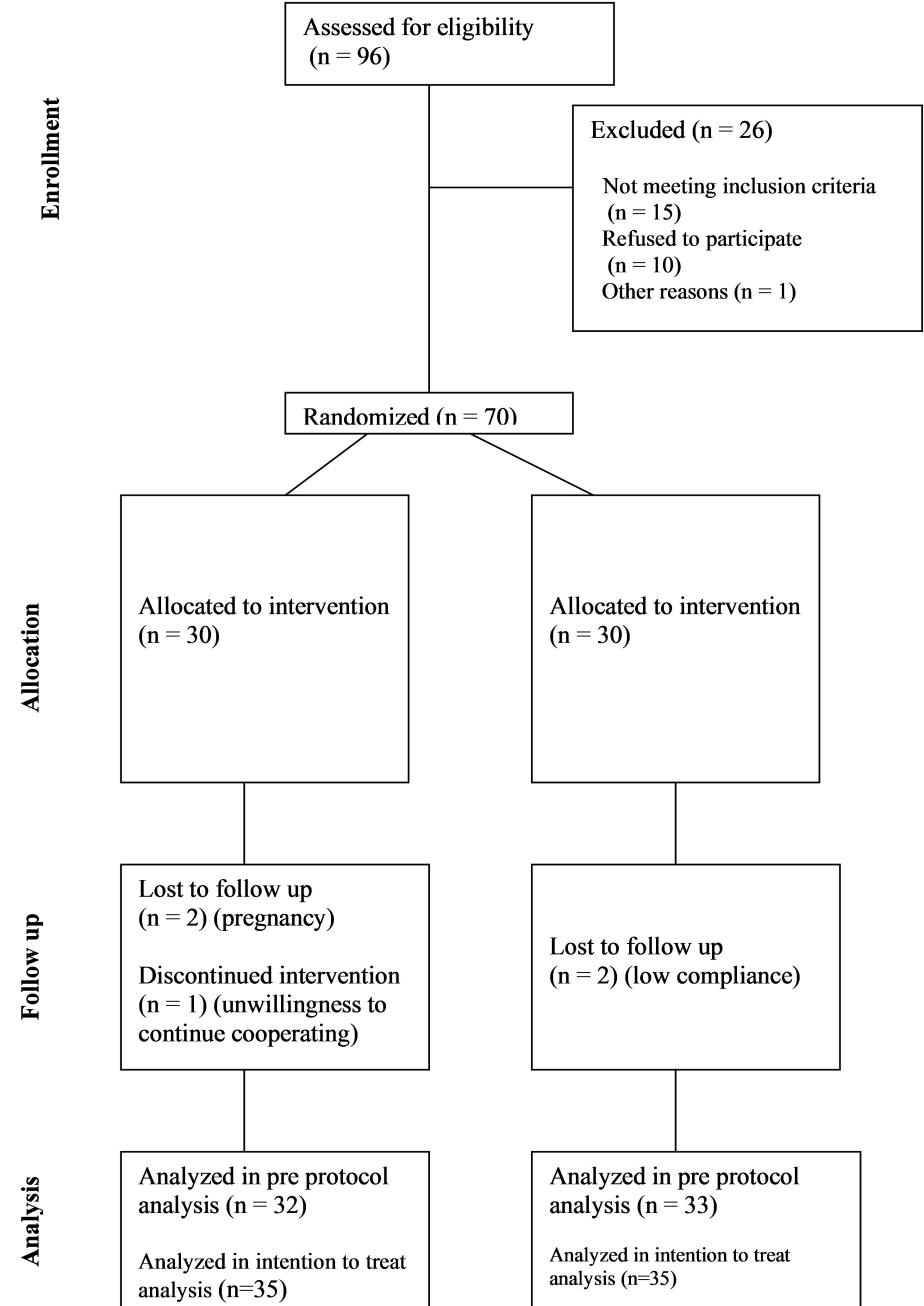
CONSORT diagram showing the flow of participants through each stage of this randomized trial.

Among the 96 screened patients, 70 were allocated to the flaxseed and control groups, and 65 completed the study (**Figure 1**). The intention-to-treat (ITT) analysis included all 70 randomized participants, whereas the per-protocol (PP) analysis considered 33 participants in the flaxseed group and 32 in the control group. In the PP analysis, two participants were excluded due to low compliance with flaxseed consumption (<90%), two due to pregnancy, and one participant withdrew from the study. **Table 1** summarized the baseline demographic and clinical characteristics of participants.

**Table 1:** Baseline characteristics of the study participants, including demographic, anthropometric, and biochemical parameters at the beginning of the trial.

Characteristics	All (n=70)	Flaxseed (n=35)	Control (n=35)	p value
Age (years)	27.43 ± 7.05	27.13 ± 7.12	27.69 ± 6.90	0.82
Weight (kg)	77.49 ± 16.23	76.90 ± 15.40	78.35 ± 16.55	0.67
BMI (kg/m^2^)	29.12 ± 6.87	28.77 ± 6.24	29.76 ± 7.05	0.71
HC (cm)	103.21 (95.23-117.31)	102.75 (93.45-116.24)	104.55 (95.83-119.20)	0.65
WC (cm)	90.32 (79.43-105.37)	88.23 (78.41-103.8)	93.26 (80.15-108.29)	0.23
FSH (µU/mL)	9.75 ± 12.27	9.72 ± 11.95	9.88 ± 12.45	0.87
LH (µU/mL)	8.65 ± 5.93	8.74 ± 5.62	8.57 ± 5.49	0.77
LH/FSH ratio	1.39 ± 0.76	1.35 ± 0.64	1.42 ± 0.79	0.23
Estradiol (pg/mL)	58.37 ± 24.93	57.23 ± 20.65	60.74 ± 25.82	0.29
DHEAS (µg/mL)	1.93 ± 1.12	1.86 ± 1.10	1.95 ± 1.15	0.36
Androstenedione (ng/mL)	3.67 ± 1.85	3.61 ± 1.62	3.70 ± 1.94	0.46
AMH (ng/mL)	8.12 ± 5.17	7.89 ± 4.93	8.36 ± 5.42	0.29
FBS (mg/dl)	91.43 ± 9.15	93.79 ± 9.76	89.50 ± 8.75	0.58
TG (mg/dl)	148.54 ± 25.56	157.62 ± 30.19	143.87 ± 24.98	0.36
TC (mg/dl)	162.34 ± 34.67	170.12 ± 37.54	156.23 ± 32.65	0.28
LDL (mg/dl)	86.14 ± 33.39	89.26 ± 34.57	82.68 ± 31.22	0.68
HDL (mg/dl)	37.26 ± 12.17	39.12 ± 13.85	34.76 ± 12.05	0.42
Fasting insulin (mU/L)	11.24 ± 4.39	11.55 ± 4.55	11.15 ± 4.18	0.78
METs (min/week) at study baseline	2495.67 ± 234.67	2478.22 ± 230.61	2510.37 ± 239.82	0.53
Menstrual irregularity (%)	88.4	89.2	87.9	0.72
Oligomenorrhea (%)	70.4	72.6	67.8	0.53
Hypermenorrhea (%)	2.5	1.7	3.5	0.36
Amenorrhea (%)	15.5	14.9	16.6	0.63

Data are shown as means with standard deviation, medians and interquartile range, or as percentages, as appropriate. Comparisons of baseline characteristics between the Flaxseed and the control group were performed using unpaired Student’s t test, Mann–Whitney U test, X2 test, or Fisher’s exact test, as appropriate. BMI, Body mass index; HC, Hip circumference; FBS, fasting blood sugar; HDL, High density lipoprotein; LDL, low-density lipoprotein, TC, total cholesterol; TG, triglyceride, WC, Waist circumference; AMH, anti-Müllerian hormone; FSH, follicle-stimulating hormone; LH, luteinizing hormone; DHEAS, dehydroepiandrosterone sulfate; MET, Metabolic Equivalent of Task.

At baseline, the mean age of participants was 27.43 ± 7.05 years, with no significant differences between groups in terms of age, weight, or BMI. Similarly, biochemical parameters, including fasting blood sugar, triglycerides, total cholesterol, LDL, HDL, and fasting insulin levels, were comparable between groups. The mean intake of calories, macronutrients and fatty acids are summarized in **Table 2**. Dietary intake analysis indicated no significant differences in total energy, macronutrient, and fatty acid intake between groups at the beginning of the study, and these remained stable throughout the intervention.

**Table 2:** Nutritional parameters at baseline and end of the study in both study groups.

Variable	Intervention group (n=33)	Control group (n=32)	P_1_-value
Energy (kcal) Baseline End P_2_-value	1875.35 ± 376.43 1743.44 ± 324.59 0.22	1753.24 ± 338.42 1678.45 ± 310.86 0.47	0.36 0.29
Carbohydrate (g) Baseline End P_2_-value	283.39± 71.44 259.66 ± 67.19 0.21	264.37 ± 66.41 260.32 ± 57.42 0.72	0.25 0.78
Protein (g) Baseline End P_2_-value	88.25 ± 27.19 78.44 ± 25.83 0.39	76.59 ± 20.47 69.85 ± 22.56 0.45	0.38 0.44
Fat (g) Baseline End P_2_-value	66.74 ± 23.19 57.37 ± 18.12 0.48	59.78 ± 19.52 57.36 ± 16.80 0.74	0.52 0.92
PUFA (g) Baseline End P_2_-value	17.32± 10.20 19.77 ± 9.47 0.57	15.77 ± 8.44 16.51 ± 6.90 0.68	0.49 0.37
MUFA (g) Baseline End P_2_-value	25.38 ± 8.79 22.73 ± 7.32 0.61	23.28 ± 7.36 20.17 ± 5.75 0.47	0.55 0.61
SFA (g) Baseline End P_2_-value	18.47 ± 10.36 16.18 ± 7.76 0.53	16.63 ± 7.85 15.93 ± 7.69 0.71	0.49 0.75

Data are shown as means with standard deviation, P1: between-group comparison of the variables; independent sample t-test. P2: within-group comparison of the variables; paired sample t-test. SFA, saturated fatty acid; PUFA, polyunsaturated fatty acid; MUFA, monounsaturated fatty acids.

After 12 weeks, FSH levels showed a significant increase in the flaxseed group compared to the control group (mean treatment effect: 0.87, 95% CI 0.086 to 1.75, P = 0.027). Additionally, the LH/FSH ratio significantly decreased following flaxseed supplementation (mean treatment effect: -0.341, 95% CI -0.63 to -0.08, P= 0.031). No significant differences were observed in AMH, LH, estradiol, DHEAS, or androstenedione changes between the two study groups (**Table 3**).

**Table 3:** Variations of the primary and secondary outcomes during the period of study in the flaxseed and control groups^1^.

	ITT (n=70)				PP(n=65)			
	Placebo Mean (SD)	Flaxseed Mean (SD)	Point Estimate (95% CI)	P_value	Placebo Mean (SD)	Flaxseed mean (SD)	Point Estimate (95% CI)	P_value
Primary endpoint								
FSH (µU/mL)	-0.71(2.05)	0.14(2.52)	0.87(0.086-1.75)	0.027	-0.74(2.11)	0.15(2.52)	0.91(0.087-1.84)	0.02
Secondary endpoints								
LH (µU/mL)	-0.15(2.66)	0.75(3.16)	-0.21(-0.523-0.134)	0.37	-0.16(2.67)	77(3.19)	-0.22(-0.525-0.135)	0.35
AMH (ng/mL)	-0.1(5.26)	-0.67(4.34)	0.091(-0.075, 0.243)	0.27	-0.12(6.12)	-0.70(5.79)	0.096(-0.076-0.247)	0.24
Estradiol (pg/mL)	5.65(19.23)	2.15(15.34)	-0.093(-0.326, 0.164)	0.49	6.19(20.35)	2.43(16.18)	-0.094(-0.329,0.170)	0.41
LH/FSH ratio	0.39(0.9)	-0.9(1.3)	-0.341(-0.630, -0.080)	0.031	0.43(1.18)	-1.03(1.3)	-0.346(-0.633,0.083)	0.027
DHEAS (µg/mL)	0.36(2.67)	0.028(2.12)	-0.014(-0.135, 0.13)	0.84	0.37(2.67)	0.03(2.15)	-0.016(-0.137,0.136)	0.81
Androstenedione (ng/mL)	0.56(3.17)	-0.09(1.76)	-0.009 (-0.128,0.125)	0.96	0.57(2.96)	-0.094(1.82)	-0.01 (-0.130,0.127)	0.86

CI, confidence interval; ITT, intention-to-treat; PP, per protocol; AMH, anti-Müllerian hormone; FSH, Follicle-Stimulating Hormone; LH, Luteinizing hormone; DHEAS, Dehydroepiandrosterone. 1. Means are changes before and after the control or flaxseed period for the primary and secondary endpoints for the ITT analysis, point estimates, and 2-sided 95% CIs.

Anthropometric measurements, including weight and BMI, remained unchanged between groups from baseline to study completion. Similarly, there were no significant differences in lipid profiles or fasting glucose levels at the end of the study. Analysis of dietary compliance confirmed that participants adhered to the study protocol, with no significant variations in reported dietary intake across the intervention period.

Throughout the study, no adverse events or side effects were reported. The intervention was well-tolerated, and no participants discontinued due to side effects related to flaxseed consumption.

## Discussion

Currently, there is limited research investigating the effects of flaxseed supplementation on reproductive endocrine profiles in individuals diagnosed with PCOS. This study sought to address this gap and demonstrated that a 12-week regimen of flaxseed supplementation resulted in notable improvements, particularly in FSH levels and the LH/FSH ratio.

The present study has demonstrated a significant elevation in FSH levels associated with flaxseed consumption. Recent studies indicate that patients with PCOS exhibit either normal or diminished levels of FSH within the hypothalamic-pituitary-ovarian axis (8, 30), which may contribute to impaired follicular development.

The consistently low yet stable levels of FSH in PCOS patients perpetuate the development of new follicles that fail to achieve full maturation and undergo atresia without ovulating. This disrupted folliculogenesis contributes to the characteristic endocrine abnormalities and clinical manifestations observed in PCOS (30, 31). Notably, research in animal models suggests that flaxseed consumption enhances FSH receptor expression in ovarian follicles, potentially contributing to improved follicular function (32). Furthermore, as previously noted, flaxseed serves as an abundant source of omega-3 polyunsaturated fatty acids. Recent research indicates that omega-3 may promote the expression of CYP51 in granulosa cells via the PI3K/Akt signaling pathway, subsequently facilitating the conversion of lanosterol into cholesterol, which is a substrate for estrogen (33).

A significant reduction in the LH/FSH ratio was also observed, suggesting a potential role of flaxseed in normalizing gonadotropin secretion. The decline in the LH/FSH ratio may be attributed to a reduction in LH levels or an increase in FSH levels. Previous studies have suggested that flaxseed oil supplementation can modulate LH levels and enhance sex hormone balance in PCOS patients (23, 33). For example, Musazadeh et al. (21) reported a significant reduction in FSH levels following flaxseed supplementation for more than 12 weeks, whereas our study observed an increase in FSH after 12 weeks. This divergence may also be due to differences in participant characteristics, baseline FSH levels, or the type of flaxseed product used (21). Similarly, Wang et al. (23) found that flaxseed oil, which is rich in α-linolenic acid but lacks lignans, resulted in FSH reduction, highlighting the potential role of specific flaxseed components in modulating hormonal responses (23). Again, the discrepancy with our results could be linked to variations in dietary background, metabolic status, or genetic factors affecting hormonal regulation in different populations. Studies have shown that in patients with PCOS, the LH/FSH ratio is elevated due to heightened sensitivity of the pituitary gland to hypothalamic GnRH or alterations in GnRH secretion patterns (8, 34, 35). Previous studies have suggested that flaxseed oil supplementation can modulate LH levels and enhance sex hormone balance in PCOS patients (23). However, variations in study duration, dosage, and flaxseed formulations may account for inconsistencies across studies.

While our study did not observe significant changes in AMH, estradiol, DHEAS, or androstenedione, these findings align with some studies reporting no effect of phytoestrogen supplementation on these parameters (36–42). However, conflicting findings exist in the literature, potentially due to differences in supplementation duration, dosage, and population characteristics (43).

While our study focused on FSH and the LH/FSH ratio, we recognize that testosterone and insulin could significantly influence these parameters. Previous studies investigating flaxseed supplementation have reported conflicting results for testosterone and insulin levels. For instance, Emmamat et al. observed a reduction in testosterone levels with flaxseed intervention (44), whereas other studies reported no significant changes in androgen or insulin levels (21). Including these outcomes could provide additional insights into the observed hormonal changes and their underlying mechanisms.

One key strength of our study is the implementation of a non-invasive and economically viable dietary intervention aimed at improving reproductive endocrine profiles in PCOS patients. This study is among the first randomized controlled trials to evaluate the effects of flaxseed supplementation on FSH levels and the LH/FSH ratio in women with PCOS. The 12-week duration of the intervention and the use of milled flaxseed, which is rich in phytoestrogens and omega-3 fatty acids, further enhance the significance of the study.

However, several limitations should be acknowledged. First, the open-labeled study design introduces the potential for bias, despite laboratory operators being blinded to treatment allocation. Second, the relatively small sample size and monocentric nature limit generalizability. Third, testosterone and insulin levels were not assessed, although these hormones could play a crucial role in modulating gonadotropin dynamics. Future research should incorporate these parameters to provide a more comprehensive understanding of flaxseed’s effects on PCOS.

## Conclusion

Our findings suggest that flaxseed supplementation, rich in omega-3 PUFAs and phytoestrogenic lignans, may serve as a beneficial dietary intervention for managing hormonal imbalances in PCOS patients. The observed increases in FSH levels and reductions in the LH/FSH ratio indicate a potential role for flaxseed in improving follicular development and ovulatory function. Further randomized controlled trials with larger sample sizes and extended follow-up periods are warranted to confirm these findings and explore the long-term impact of flaxseed supplementation on reproductive outcomes in PCOS patients.

## Data Availability

The raw data supporting the conclusions of this article will be made available by the authors, without undue reservation.
