# Supplementary material for: Flaxseed intervention and reproductive endocrine profiles in patients with polycystic ovary syndrome: an open-labeled randomized controlled clinical trial
Source: Front Endocrinol (Lausanne). 2025 Apr 7;16:1531762. doi: 10.3389/fendo.2025.1531762 (PMC12010250; doi:10.3389/fendo.2025.1531762)
Supplement: Supplementary file 1 [file DataSheet1.pdf]

24-Hour Nutrition Recall Form

Study Title: Flaxseed Intervention and Reproductive Endocrine Profiles in Patients with Polycystic Ovary Syndrome
Participant ID: ____________
Date: //____
Interviewer Name: ____________

Instructions:

This form is designed to record all food and beverage intake over the past 24 hours. Please provide detailed information, including portion sizes, cooking methods, and any condiments or seasonings used. If unsure, provide the best possible estimate.

Section 1: General Information

1. **Did you have a typical eating day yesterday?** (Yes / No)
   - If no, explain why: ________________________
2. **Time of first meal/snack:** ____________
3. **Time of last meal/snack:** ____________
4. **Physical activity performed in the last 24 hours (type and duration):** ________________

Section 2: Food & Beverage Intake Record

Time	Meal/Snack	Food/Beverage	Quantity/Portion Size	Cooking Method	Condiments/Seasonings
08:00 AM	Breakfast	Scrambled eggs	2 eggs	Fried with oil	Salt, pepper
10:30 AM	Snack	Apple	1 medium	Raw	None
01:00 PM	Lunch	Grilled chicken breast	100g	Grilled	Lemon juice, olive oil
04:00 PM	Snack	Almonds	10 pieces	Raw	None
07:30 PM	Dinner	Brown rice with vegetables	1 cup	Boiled	Olive oil, salt
10:00 PM	Snack	Green tea	1 cup	Hot water	Honey (1 tsp)

(*Note: The table above is an example. Participants should fill in their actual intake.*)

Section 3: Additional Details

1. **Did you consume any supplements (e.g., vitamins, minerals, omega-3, protein powders)?**
    - Yes / No
    - If yes, please specify: _______________
2. **Did you consume any packaged or processed foods?**
    - Yes / No
    - If yes, please specify brand and type: _______________
3. **Did you eat out or prepare all meals at home?**
    - Home / Restaurant / Other (specify): _______________

Section 4: Researcher Notes

(For interviewer use only – any observations, missing details, or additional notes)

Footnote:

(*the original version was in Farsi and has been translated for accessibility*.)

Thank you for your participation!
